# Experiences of Participants in a Self-Management Program for Employees with Complaints of the Arm, Neck or Shoulder (CANS): A Mixed Methods Study

**DOI:** 10.1007/s10926-016-9630-9

**Published:** 2016-02-13

**Authors:** Nathan Hutting, Sarah I. Detaille, Yvonne F. Heerkens, Josephine A. Engels, J. Bart Staal, Maria W. G. Nijhuis-van der Sanden

**Affiliations:** 1 Radboud Institute for Health Sciences, IQ Healthcare, Radboud University Medical Center, Nijmegen, The Netherlands; 2 Research Group Occupation and Health, Faculty of Health and Social Studies, HAN University of Applied Sciences, PO Box 6960, 6503 GL Nijmegen, The Netherlands; 3 HAN Seneca, Expertise Centre for Sports, Work and Health, HAN University of Applied Sciences, Nijmegen, The Netherlands; 4 Research Group Musculoskeletal Rehabilitation, Faculty of Health and Social Studies, HAN University of Applied Sciences, Nijmegen, The Netherlands

**Keywords:** Musculoskeletal pain, Qualitative research, Cumulative trauma disorders

## Abstract

*Purpose* To investigate the experiences of participants of a self-management program for employees with complaints of the arm, neck or shoulder (CANS). The program consisted of six group sessions combined with an eHealth module. *Methods* Semi-structured interviews with the first 31 consecutive participants of the intervention group participating in a randomized controlled trial. Participants were interviewed after their last group session. Semi-structured interviews were guided by an interview guide and audio-recorded. Data were analyzed using thematic analysis and the emerging themes were discussed. All participants in the intervention group were asked about their experiences with a questionnaire at three (n = 58) and 12-months (n = 53) follow-up. *Results* Most participants appreciated the diversity of the program and benefited from the interaction with their peers. The eHealth module, although not used by everyone, was generally experienced as positive, especially the section with the physical exercises. Participants obtained more insight into their complaints and increased awareness, which contributed to the acceptance of and coping with the complaints. There was also criticism about the content of the program and the lack of a follow-up session. Results of the questionnaires showed that participants had a high level of satisfaction. *Conclusions* In general, the intervention fitted the needs of employees with CANS. Participants obtained more knowledge and insight into their complaints, as well as increased awareness; all this contributed to a behavioral change and improved coping. Many participants made changes at work and during their leisure time, whereas some felt that continuing their ‘changed’ behavior would be a challenge.

**Electronic supplementary material:**

The online version of this article (doi:10.1007/s10926-016-9630-9) contains supplementary material, which is available to authorized users.

## Introduction

Chronic musculoskeletal pain is a worldwide health problem resulting in negative effects on an individual’s wellbeing, as well as costs to society [1]. Most common musculoskeletal problems include osteoarthritis, rheumatoid arthritis, and spine-related neck and back problems [1–3]. Work-related musculoskeletal disorders are a substantial problem in the workplace, leading to human suffering, lost time due to sickness absence, and lower work productivity [4]. Work-related musculoskeletal disorders are frequently underreported at the workplace as many employees attempt to continue to work despite having complaints [5, 6]. Complaints of the arm, neck or shoulder (CANS) [7], also known as work-related musculoskeletal upper extremity disorders [8], represent a high proportion of work-related musculoskeletal disorders [9]. Moreover, CANS are persistent [10] and 58 % of the people suffering from chronic complaints, such as CANS, report the use of healthcare e.g. care given by the general practitioner, medical specialist and physical therapist [11]. CANS have a multifactorial origin [12–15], including physical characteristics, psychosocial characteristics, personal factors, and environmental factors [10, 12–20]. The importance of each factor, and its individual contribution to the risk of provoking symptoms, vary among individuals and work environments [21].

Employees with work-related musculoskeletal disorders, including CANS, are faced with the challenge to deal with their complaints on a daily basis in both their private and working life [22, 23]. Employees with CANS are generally not fully aware of the possibilities to influence their symptoms and their own role in triggering and coping with their complaints, and often go beyond their individual limits [22, 24]. Moreover, employees need to become aware of the causes of their complaints and realize that they need to take action [22–24]. Although many employees with CANS try different therapies and self-treatments to reduce their complaints, they often still suffer from complaints [22, 23].

A recent Cochrane review on conservative interventions for treating work-related CANS, found that exercise, ergonomic intervention, or behavioral intervention generally had no consistent effects on the outcome measures (e.g. pain, recovery, disability), compared to no treatment, other treatment, or placebo treatment [25]. Thus, there seems to be a need for effective intervention programs for people with CANS [7, 25, 26]. Given the multifactorial (bio-psychosocial) origin of CANS, multi-component personal-tailored interventions that include both biomechanical and psychosocial components are recommended [8, 14, 27].

Because of the worldwide burden of chronic conditions, including chronic pain, promoting and improving the way patients self-manage their conditions is recognized as important [28]. Following the intervention mapping protocol [29] we adapted an existing generic self-management program for employees with a chronic somatic disease developed by Detaille et al. [30, 31] and added an eHealth module for use in employees with CANS [32]. The effectiveness of the adapted intervention for employees with CANS was examined in a randomized controlled trial (RCT) [33, 34]. On the Disabilities of the Arm, Shoulder and Hand (DASH) questionnaire work module (which evaluates the impact of arm, shoulder or hand problems on the participants’ ability to work) the intervention group (self-management sessions and an eHealth module) as well as the usual care group (receiving usual care only for CANS), showed clinically relevant improvements; however, the intervention group showed a significantly better improvement compared to the usual care group (*P* = 0.04) over a 12-months period. Moreover, 12 months after the start of the intervention, the limitations experienced in work-related activities in the intervention group had decreased significantly compared to the usual care group (*P* = 0.04). The control group had a significantly higher number of mean hours performing sport activities in the previous 3 months compared to the intervention group (measured at 12 months), indicating that they had changed their behavior with regard to sport activities. None of the other measured outcomes showed a significant difference between the groups [34].

Together with the RCT, a mixed methods evaluation, with the participants in the intervention group, was planned to investigate whether the developed self-management program and program topics fitted the needs of employees with CANS. This article presents the results of this evaluation.

## Methods

### Participants

The first 31 consecutive participants of the intervention group of the RCT were invited by the first author (NH) for a semi-structured interview. Participants were included until saturation was reached. The point of saturation was defined as the point at which no new codes were added during three consecutive interviews during data analysis.

Furthermore, all participants in the intervention group of the RCT were asked about their experiences with the intervention in the three and 12-months follow-up questionnaires.

All participants gave written informed consent to participate in the study and to allow audio-recording of the sessions. The Medical Ethics Committee of the Radboud university medical center (located in Nijmegen, the Netherlands) approved the study design, protocols and procedures (registration number 2012/319).

### Self-Management Intervention

The self-management intervention for employees with CANS consisted of six group sessions (5–10 participants) of 2.5 h each led by a moderator (AN, EN, IB, NN, SD), combined with an eHealth module. The eHealth module was available for 1 year. An overview of the content of the program is presented in Table 1. The development and the content of the self-management intervention are described elsewhere [32, 33]. Action plans were made during the sessions. Action planning is an important component of self-management interventions, with successful completion being associated with improved health and self-efficacy outcomes [35].

**Table 1:** Topics of the group sessions and eHealth module

Topics of the group sessions	
Session 1	Introduction
	Dealing with a chronic disability
	Living with CANS
	Working with CANS
	Work load and work capacity
	What is self-management?
	Introduction to the eHealth module
Session 2	Discussion on the eHealth module
	Core qualities
	Time management
Session 3	Dealing with pain and fatigue
	Stress and stress management
	(Muscle) relaxation exercises
Session 4	Healthy lifestyle
	Nutrition
	Exercises and sports
	Use of facilities
Session 5	Communication skills
	Working with others and asking for help
Session 6	Dealing with negative emotions
	Positive thinking
	Making a mind map

Topics of the eHealth module	
Topic	Content
Use of eHealth	Manual of the eHealth module
Self -management	Introduction to self-management
CANS	Non-specific CANS, specific CANS, symptoms, causes (workload and capacity, physical factors, psychosocial and personal factors, chronic pain, sensitization, self-tests and screening tests), prognosis
Possible solutions	What can I do? (workplace, work pressure and work style, reduction of stress, sports and specific exercises), facilities within organisation, treatments
About the group sessions	Topics of the group sessions and manual
Further reading	Additional information and references to websites

### Data Collection

Characteristics of all participants were collected before the start of the self-management sessions. Participants were interviewed by the first author (NH), mostly in the first 3 weeks after the last group session. All semi-structured interviews were guided by an interview guide (Appendix 1). The interview guide was developed by the authors and focused on the participants’ reasons to participate, and their expectations, benefits, future expectations and experiences with the action plans, group sessions and the eHealth module. Participants were also asked how the intervention could be improved. All interviews were audio-recorded.

All participants in the intervention group received a digital questionnaire asking about their experiences with the self-management program at three and 12-months follow-up. This latter questionnaire was offered together with questionnaires regarding the outcome measures of the quantitative evaluation.

### Data Analysis

**Fig. 1:**
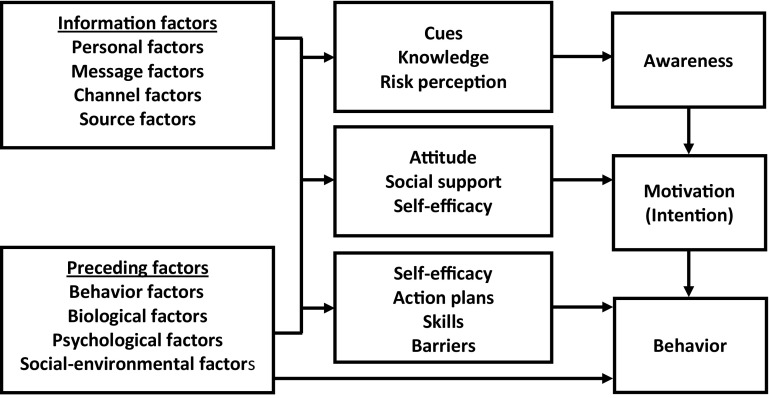
The I-Change model (2.0) [38]

The audio-recordings were fully transcribed by an assistant (LD). Respondent validation was performed by emailing the transcription of the interview to the participant. Participants were asked to check the transcription for errors and misinterpretations. If no response to the first email was received from participants within 10 days, a reminder was sent by email. Two authors (NH, SD) trained in qualitative research methods performed the data analysis. Data were analyzed using theoretical thematic analysis, a method for identifying, analyzing and reporting themes within data [36]. Analysis was performed by taking the following steps: (1) familiarizing with the data; (2) generating initial codes; (3) searching for themes; (4) defining and naming themes; and (5) producing the report [36]. The first three transcriptions were analyzed by both authors; thereafter, the codes emerging from the data were compared and discussed until consensus was reached. The subsequent interviews were analyzed by one author (NH) and randomly checked by the second author (SD). The emerging themes expressing the perceived effects of the intervention were presented according to factors of the I-Change model (2.0) [37]. The I-Change model builds on the Attitude-Social influence-Efficacy model [38] (comparable to the theory of planned behavior [39–41]) and integrates ideas from several social cognitive models [37]. The I-Change model assumes that the behavioral change process can be distinguished in three phases: (1) Awareness; (2) Motivation; and (3) Action [42] (Fig. 1). Because our previous focus group study showed that the emerging themes had similarities with the I-Change model [24], we decided to cluster the emerging themes according to this model.

The Atlas.ti (version 7.1.8), Scientific Software Development GmbH program was used for analysis. During data analysis, the emerging themes were discussed by two authors (NH, SD). The supporting quotes related to each theme were discussed by all authors.

The results of the quantitative evaluation of the experiences of all participants in the intervention group at three and 12 months were displayed as the percentage of participants for every response option; analyses were performed using IBM SPSS Statistics 20.

## Results

### Participants

In the present study, 31 participants of the self-management intervention were interviewed. Four of these 31 participants did not complete the self-management sessions. However, three of the four continued to use the eHealth and were willing to participate in this study, and one refused (total n = 31). The mean age of the participants was 46.1 (27–61) years. The mean duration of complaints was 19.9 (12–650) weeks, and 16 participants (51.6 %) received treatment for their complaints in the previous 3 months. The demographic profile of each participant is presented in Appendix 2.

At baseline, the mean age of the total intervention group (n = 64) was 45 (SD 11) years. In the quantitative evaluation at three and 12 months, 58 (92 %) and 53 (82 %) participants, respectively, filled in the questionnaire with regard to their experiences with the program.

### Data Analysis

In total 31 participants were interviewed, after which data saturation was reached. Interviews lasted 10–23 min (excluding the introduction time). All participants were reached for the respondent validation in which they could comment on the transcription of their interview. Seven participants had small comments, all of which were processed in the transcription. Both authors performing the data analysis agreed on the codes that emerged. The themes emerging during data analysis are described below. We discuss in succession: (1) expectations of participants; (2) experiences with the program; (3) perceived effects of the intervention; (4) reasons for drop-out; and (5) practical recommendations.

### Expectations of Participants

Some participants had no clear expectations, whereas others were simply curious and had an open mind. Some wanted to participate without any expectations about possible benefits from the program, i.e. they thought that the program would at least be interesting or beneficial for them. Most participants did not expect a ready-to-use solution. The aspect of raising awareness and how to translate this awareness into action, was an expectation. However, two participants expected some immediate results after the intervention, and most indicated that they did not expect to get rid of their complaints. Some participants expected to exchange experiences with others in the sessions. Other expectations included to acquire skills/tools on how to cope/deal with their symptoms, and to define one’s own limits. Especially tools for long-term self-management of their complaints were expected.

#### Meeting the Expectations

The program met the expectations of most participants and sometimes even exceeded expectations. This was supported by the results of the questionnaire provided at the 12-months follow-up (Table 2, question 8), indicating that the intervention ‘totally’ or ‘somewhat’ met the *expectations* of 66 % of the participants. Moreover, the intervention ‘totally’ or ‘somewhat’ met the *needs* of 68 % of the participants (Table 2, question 7). Participants learned new things and the program provided a valuable perspective. Moreover, the program created awareness and facilitated the exchange of experiences with other participants. Participants recognized their personal characteristics in the content and examples of the self-management meetings. One person stated:

**Table 2:** Experiences of participants in the intervention group

Statements (3 months), n = 58	1 (%)	2 (%)	3 (%)	4 (%)	5 (%)
1. The content of the self-management sessions was generally useful for me	39.7	37.9	10.3	3.4	8.6
2. The moderators were capable of moderating the self-management training	62.1	25.9	5.2	3.4	3.4

Questions (12 months), n = 53	A (%)	B (%)	C (%)
3. Has the intervention played a role in your considerations to visit a physician for your complaints?	3.8	17.0	79.2
4. Has the intervention played a role in your considerations to visit a therapist for your complaints?	7.5	34.0	58.5
5. Has the intervention played a role in your considerations to ask for an ergonomic workplace investigation for your complaints?	5.7	20.8	73.6
6. Has the intervention played a role in your considerations to (let) adapt your workplace?	11.3	30.2	58.5

Statements (12 months), n = 53	1 (%)	2 (%)	3 (%)	4 (%)	5 (%)
7. The intervention met my needs.	18.9	49.1	18.9	11.3	1.9
8. The intervention met my expectations.	15.1	50.9	17.0	17.0	0.0
9. The intervention played a major role in reducing my complaints.	13.2	39.6	22.6	17.0	7.5
10. I would recommend the intervention to colleagues with CANS.	37.7	37.7	15.1	5.7	3.8
11. I am capable of what I have learned in the intervention to apply in practice.	17.0	66.0	11.3	5.7	0.0
12. The eHealth module was a good addition to the self-management sessions.	18.9	41.5	30.2	1.9	7.5
13. The information provided in the eHealth module was generally useful for me.	18.9	47.2	20.8	5.7	7.5

*1* totally agree, *2* somewhat agree, *3* neither agree nor disagree, *4* somewhat disagree, *5* totally disagree

*A* Yes, to a large extent, *B* Yes, to some extent, *C* No

For me—it exceeded my expectations. Many more factors are involved than just a wrong posture. (…) Now I’m much more aware of the causes of CANS–for example, if I have stress this aggravates the complaints … Of course posture is important, but so are all those other factors. It’s given me more than I could expect. (participant 3)

#### Not Meeting the Expectations

Some participants expected the program to focus more on the physical component. Moreover, some expected the involvement of a physical therapist (e.g. to make a thorough physical examination) and expected more information on physical components, especially with regard to posture and physical activity. Others expected exercises for their complaints in the self-management sessions and were disappointed. One participant stated:

The program didn’t meet my expectations at all. It was very psychologically orientated … it could have been more focused on the physical aspects of the complaints. (participant 6)

Most participants who expected a more physical approach recognized the value of the bio-psychosocial approach of the program. One participant who expected more involvement of a physical therapist stated:

I expected a more conventional physical therapy approach. But, looking back, I think the balance in the program was very good. (…) The approach is very broad. I do agree … that all those factors influence the complaints. (participant 4)

Other participants who had a more physically-orientated expectation of the program agreed with that view, and were very satisfied that the program was so diverse, addressing both physical and psycho-social aspects. One lady (participant 5) who had also expected the program to be more focused on the physical aspects of CANS, became aware that a physical approach was not what she needed because she had already tried many physically-orientated therapies or adaptations. Some indicated that the program only partly met their expectations because they were already very experienced in dealing with their complaints and had not heard many new things. However, mainly because of the mutual social support and learning from each other, the program was still experienced as being valuable for them.

### Experiences with the Program

In general, participants were very satisfied with the program. About 78 % ‘totally’ or ‘somewhat’ agreed that the content of the self-management sessions was generally useful for them (Table 2, question 1). Moreover, about 75 % ‘totally’ or ‘somewhat’ agreed that they would recommend the intervention to colleagues with CANS (Table 2, question 10). Participants benefited from the program, as indicated by the following comment:

I’m very positive about the program, it was very useful. Eventually, it’s all about having to do it yourself. Now I’ve got so many skills—and a lot of information that I can use in the future. (participant 3)

About 53 % of the participants ‘totally’ or ‘somewhat’ agreed that the intervention played a major role in reducing their complaints (Table 2, question 9). Some indicated that their symptoms decreased during the program or that they were easier to manage, as one participant said:

I’m happy that I participated. My complaints aren’t totally gone, but I can control them much better. (participant 20)

Others had a short-lasting decrease in complaints or did not mention a decrease in symptoms during the program. One lady (participant 13) said that, after a period of fatigue after work, she now had more energy at the end of the day. In general, the diversity and scope of the program was seen as a strong point: the intervention felt like a ‘package’, which was experienced as a major positive point.

### eHealth Module

Participants generally found that the eHealth module was well constructed with good information and references to other sources. Participants could find their way on the website, although one lady said that she found it difficult to navigate. The eHealth module was generally reported to be very accessible. One participant said:

Yes, I really liked the eHealth. Firstly, with these kinds of complaints you’re always searching (…) for all types of reliable information. Secondly, I found the exercises beneficial—I perform them regularly. I really like them and they’re clearly explained. Those are the things I liked most about the eHealth module. (participant 20)

Participants liked the background information on the eHealth module. These additions, compared to the sessions, were considered to be valuable, e.g. the topic about workplace solutions and investigations. Most participants that did not use the eHealth module extensively planned to look at it in the future, or if their complaints became worse. Many participants only looked at it a few times; generally, about half of the participants did not use the eHealth module extensively. This was mainly due to lack of time or because they did not find it interesting. Others said that they did not want to spend more time on their computer, or that it did not add much to the sessions. The results of the questionnaire indicated that about 60 % of the participants ‘totally’ or ‘somewhat’ agreed that the eHealth module was a valuable addition to the self-management sessions (Table 2, question 12) and about 66 % ‘totally’ or ‘somewhat’ agreed that the information provided in the eHealth module was useful for them (Table 2, question 13).

Some participants felt that the eHealth module and the sessions partly covered the same topics, others found that the eHealth module and the sessions complemented each other and found the eHealth module to be a worthwhile addition, whereas others merely indicated that they benefited from the sessions and the eHealth module.

### eHealth: Exercises

The exercises of the eHealth module were generally very useful for almost all users and were rated very positively. Some stated that the exercises were well filmed and explained, and facilitated taking action. One participant would have liked a printable version of the exercises, others used the eHealth module only for the physical exercises. Some indicated that the exercises were the tools that they benefited most from, as one participant said:

I looked at all the exercises in the eHealth module the first day. They’re really useful and easy to perform—I try to do them regularly. Now I know that I can do these exercises by myself, this was the support I needed. (participant 27)

### Group Sessions

Participants liked their group size (5–6 participants per group). The small size made interaction easy and participants felt safe; there was a pleasant atmosphere. One lady (participant 22) preferred a slightly larger group as this might have been more interesting, but also realized that this would have involved more time; this latter group sometimes had only four participants (due to drop-out and illness) and another person (participant 23) in this group said that six participants might have been ideal.

Participants who mentioned the moderator were positive about the moderator: 87 % of the participants ‘totally’ or ‘somewhat’ agreed that the moderators were capable of moderating the self-management sessions (Table 2, question 2). Participants liked the professional and personal attitude of the moderators; they were able to personalize the session content and created a personal atmosphere. Some mentioned that some of the topics were discussed too extensively by the moderator.

Participants mentioned that the session with the human movement scientist/physical therapist was very valuable and interesting; they liked receiving information about muscle function, and the influence of exercises and training. Some participants had expected more personalized advice and exercises, although they confirmed that it was possible to ask personal questions. Some had expected that exercises would also be performed in this particular session. Others mentioned that this session with the therapist could have been more extensive. One participant said:

I really liked that session, he also performed some relaxation exercises with us. That physical part—that could have been discussed more extensively. (participant 26)

### Perceived Effects of the Intervention

#### Awareness

Participants stated that they experienced increased awareness during the intervention, which was considered very valuable. Several participants stated that this increase in awareness was the most important effect of the program. One lady (participant 2) said that she already had a high level of awareness, but the recurrence and endorsement were very valuable. Another participant said:

I became more aware that I have to do something about my complaints myself. It’s not something that will heal itself—you really have to be actively involved. It is something of great importance for the rest of my life—that I always have to remember that I should chill out. I’m more aware now of the situations which produce stress for me—so I try to deal with them. (participant 15)

For some participants the intervention was a confirmation of thoughts they already had, which strengthened them in their beliefs. One participant said:

I really benefited from the program. What have I learned? Mainly awareness. Awareness of the chronic character of the complaint- that it can come back from time to time. More insight into the causes of the complaints. Insight into things I can do to deal with and decrease my complaints—but awareness is the most important for me. (participant 20)

Participants understood and recognized themselves and their group members as a special type of person, who feels very responsible and who are at increased risk to develop CANS. Some participants also became aware about personal characteristics, e.g. their tendency for perfectionism.

Awareness that the complaints could be related to their own behavior stimulated participants to listen to their body signals and pay attention to the role of work stress and their own work style, and to the need of taking action.

#### Knowledge and Insight

Participants mentioned that the program provided knowledge about their complaints and insight into their complaints. Some participants gained more insight into the factors that provoked/aggravated their complaints. They realized that many factors (e.g. stress) may contribute to the origin and persistence of their problems. Some already knew that all these factors were involved, whereas for others this was a new insight. The information about central sensitization was valuable for some participants and contributed to insight into their complaints and the process of acceptance. However, there was also some resistance to the principle of central sensitization, mainly because of the complicated concept and the interpretation of some participants that their complaints were ‘not real’.

### Motivation to Change

#### Attitude

Some participants said that they changed their attitude towards their complaints. For example, one lady (participant 2) said that she changed how she looked at her complaints because she realized that she was not the only one with complaints, and there are always others with worse symptoms. Another lady (participant 4) stated that the most important change was the way she looked at the pain, i.e. she no longer saw it as a sign of tissue damage.

Some participants were not aware that their complaints might be chronic and that it is important to cope with the problems at work and in daily life. Although it was perceived as difficult to accept that the complaints may never disappear completely, most found it useful to realize that the complaints might be chronic and that they should learn to cope with these problems. One participant who found this difficult, said:

It was very painful to realize that the complaints could be chronic and might not go away. I found that very difficult. Perhaps you’d think that after so many years of complaints, I should know that—but it was confrontational and required a change of mind to accept it and make a plan to deal with it. (participant 30)

Acceptance of and coping with complaints were frequently mentioned as a useful effect of the intervention and resulted in a changed attitude towards their complaints. One participant said:

During the sessions I came to some sort of acceptance—I have to cope with my complaints and I just need to try and keep them manageable. That’s what the course has accomplished. (participant 10)

#### Social Support

Participants liked the interaction between participants; they could learn from each other and felt supported. Exchange of experiences was rated very positively. One participant said:

It was very useful to hear the experiences of the others and get a lot of information. At certain times you feel alone with your complaints, even though you know many people suffer from CANS. I benefited from the recognition of the complaints by other participants and hearing how others deal with the complaints. (participant 18)

Participants liked the fact that they differed from each other; they could hear different stories and advice, and place their own problems in a better perspective. On the other hand, the interaction and telling each other their stories during the sessions took a lot of time and was not valuable for all participants. In general, participants felt very secure and safe in the group. With regard to the support received from supervisors, colleagues, and family and friends, the intervention group experienced this as generally being high (see Table 3).

**Table 3:** Experienced support of participants in the intervention group at 3 months (n = 53)

Experienced support of participants in the intervention group at 3 months (n = 53)	Median	Range
Statement (7 point Likert scale)		
*1* = *Very much opposition* *7* = *A lot of support*		
To participate in the intervention I experienced from my supervisor	5	2–7
To participate in the intervention I experienced from my colleagues	5	2–7
To participate in the intervention I experienced from my family and friends	6	2–7
In achieving my personal goals, I experienced within my organization	5	2–7
In achieving my personal goals, I experienced from my supervisor	4	1–7
In achieving my personal goals, I experienced from my colleagues	4	1–7

### Behavior

Participants became more aware that they should learn to cope with the complaints and change their behavior; they were motivated to really take action. Participants mentioned that they changed their behavior, e.g. at work, at home, and with regard to sport activities. On the questionnaire, 83 % of the participants totally’ or ‘somewhat’ agreed that they were able to apply in practice what they had learned in the intervention. Participants said that they were more aware and had adapted their lifestyle and performed exercises. One participant said:

I have changed totally. (…) I’ve just walked outside. Before the intervention, I didn’t do that. (…) Also awareness about taking breaks - just go outside, walk over to a colleague, or drink a cup of coffee. That’s what I’m doing… and taking the stairs instead of the elevator. I also didn’t do that before. (participant 5)

However, some participants mentioned that they knew beforehand that it would be difficult to change their behavior and habits; it is easy to fall back into old habits, also at work. The threshold to take action was lowered during the program and the participants stimulated each other, for example:

You’re confronted with the facts again—and you’re more actively involved. (…) You’ve made your action plan which you try to realize every day. (participant 14)

#### Skills

Many participants became more active in daily life and performed sport activities, including Pilates, yoga, fitness, or swimming. One participant (participant 4) said that she had not yet succeeded in playing tennis again, partly due to her fear of increasing pain. Moreover, many participants performed exercises at home and some were stimulated to search for care for their complaints, e.g. physical therapy. Participants said that they were also stimulated to be more physically active, e.g. cycling to work, or taking the stairs instead of the elevator, or walking during their breaks. Table 2 presents the results of the questionnaire on the extent to which the intervention played a role in participants’ consideration to visit a physician or physical therapist, or to ask for an ergonomic workplace investigation and/or adaptation of their workplace (questions 3–6).

Also, many participants indicated that they had changed their work style and realized that they should take breaks, which they did now. However, some participants still found it difficult to take breaks:

If I want to take breaks, I really have to schedule them in my agenda—but I still don’t always take them. But when I look back I think, that half an hour doesn’t really matter. (participant 23)

Some participants were more aware of their own limits, and set those limits after participating in the program. Others indicated that they were more able to let things go, as shown by the following quote:

I really benefited because I’m the kind of person that never said ‘no’ but always said ‘yes I’ll do it’. But I don´t do that anymore—that resulted in less stress and I’m now better at delegating tasks. (participant 8)

Some participants said that they communicated more: for instance, if they were irritated by something, they now mentioned it. One participant said:

What I do now is make things negotiable… I didn’t do that in the past, I kept it all to myself. (participant 25)

Also at home, participants made some modifications and adapted their behavior. Sometimes they also involved their partner and changed their lifestyle together. Some participants felt more relaxed and, e.g., divided household chores over two days (participant 7). One participant also became aware that there are more things in life than work, as she said:

(…) I also have to do things I like, and not just the things I need to do. That’s something I became aware of again. I do have some leisure time, but I also make obligations for myself. That’s something I had to stop…. (participant 13)

#### Action Plans

In general, participants experienced the action plans they had to make and carry out during the sessions as a helpful tool in taking action. The action plans were seen as an incentive to take action. Explicating the planned behavior was a useful pathway to making changes and some had already made some kind of action plans themselves. One lady (participant 28) said it was stimulating that the moderator also made her own action plans. In general, participants were aware that continuing their changed behavior is the next step to success.

Participants were also aware of the importance of making concrete, manageable, and SMART action plans. During the process, this was getting better and better. Although most participants had no problem with the execution of their action plans, some did (mainly due to limited time or to the prioritization of other things first). Discipline is considered very important, one participant said:

… mainly self-discipline. (…) There were some things I intended to do, which at first were either not done or were done later. But eventually I got things on the rails - so it worked well. (participant 9)

#### Self-Efficacy

Participants were looking to the future with confidence, although some stated that it is important to continue working at their complaints in order to control them. Some mentioned that the last session, in which the future was discussed and a mind map was made, was valuable and interesting. Some also believed that their complaints will go away in the future, while others thought that some symptoms will persist. Some participants with a physically challenging job had questions about their future, although one of them explicitly said that she intended to stay actively involved in handling her complaints, which was endorsed by other participants. One of them stated:

It’s not something that comes to an end after six sessions—it’s something you have to continue working at. (participant 8)

Some participants indicated that it will not be easy to continue their behavior; this was endorsed by a participant who was not very confident about the future:

No—and that sounds very negative, And that’s not how I want to see it—but I hope I’ll think about the course and benefit from it, especially if my complaints become worse. I think I can benefit from it for a long time, but I have the feeling that I’ll fall back into old patterns quickly—unfortunately. (participant 11)

#### Barriers

Participants mentioned some barriers to changing their behavior. For example, one lady (participant 7) said that, especially at work, it was not always easy to change her behavior, because it also depends on environmental l factors, such as the availability of colleagues. Most action plans were related to personal factors, but some participants made action plans which were also related to environmental factors, although not all environmental factors were manageable. For example, one lady (participant 1), who experienced difficulties with the air conditioning at her workplace did not succeed in finding a solution for this problem. However, in general, participants experienced cooperation from their supervisor to realize their action plans. To continue performing the changed behavior and to continue using action plans were considered important, but not always easy.

#### Reasons for Drop-Out

The three interviewed drop-outs of the self-management sessions participated in 1-3 sessions. One participant (participant 26) said that she stopped because of limited time, especially the time needed for the session combined with the travelling time. She specifically mentioned that her drop-out had nothing to do with the content of the sessions. Another participant also mentioned lack of time as a reason for dropping-out. Although participants who missed one or more sessions were given the opportunity to follow these in another group, this particular participant chose to continue using only the eHealth module and was happy that she could use that for a year. Moreover, she said that a more extensive eHealth program, without the sessions, might have been better for her. Another drop-out had a totally different reason:

I was embarrassed about myself—that I didn’t have the discipline and take the responsibility to change the things that needed to change. If I really want to do this right then I have to change a lot: do sports, (…) get more rest, take more breaks at work, do more exercises, don’t sit so much behind the computer at home… (…) This also discouraged me. (participant 26)

#### Practical Recommendations

Participants gave practical recommendations for improvement of the program. In one group the participants performed exercises during the sessions because they felt they had to sit too long. Moreover, some would prefer to see more focus on the physical part of the complaints in the sessions, including more exercises. One participant said that for her the sessions could have been more compatible with the text of the manual. Some would like the sessions to take less weeks, or be clustered together in less days, while others wanted more sessions (e.g. for 8 weeks).

Several participants said that they would like a follow-up session after some weeks or months, or even some follow-up sessions every 6 months; another participant said that an online community might be valuable. A follow-up could serve as a stimulus to retain the changed behavior. Some groups made a follow-up appointment with the groups themselves.

The topic about nutrition was mentioned several times as being redundant, although some found this a useful, non-obvious, topic. The topic about communication (with regard to the employer) was once mentioned as redundant.

With regard to the eHealth module, one participant said that more pictures would have been helpful. Another recommendation was a page containing details of the sessions. One participant would have preferred a total e-version of the intervention. In addition, use of the eHealth during the sessions, quicker loading of the movies of the exercises, and a more modern look were mentioned.

## Discussion

In this study, semi-structured interviews and questionnaires were used to investigate the experiences of participants of a self-management program combined with an eHealth module, for employees with CANS. The results of this study provide insight as to whether the program fitted the needs of employees with CANS, into the success factors of the program, and into factors which might need adaptation.

In general, the interviews revealed that participants were satisfied with the program and with the diversity and wide scope of the program, although some participants would have preferred more focus on the physical diagnosis and intervention. In almost all participants, a behavioral change was facilitated. Many participants made changes at work and in their leisure time, but some also felt that continuing their changed behavior would be a challenge. The results of the semi-structured interviews are supported by the results of questionnaires filled in at three and 12 months. Generally, it can be concluded that the intervention met the needs and expectations of participants and that they were very satisfied with the program.

The perceived effects of the intervention are related to the phases of the I-Change model (2.0, i.e. awareness, motivation and action) [37], a model that is built on the Attitude-Social influence-Efficacy model. The intervention was also developed using the Attitude–Social influence–Efficacy model [38]. Raising awareness was experienced as a major effect of the program, an item that was also mentioned after self-management programs for employees with a chronic somatic disease [43] and for heart failure patients [44]. It is possible that participants in the present study obtained knowledge and insight into their complaints which, together with increased awareness, contributed to the acceptance of and coping with their complaints. Participants were motivated and sometimes changed their attitude towards their complaints.

In the interviews, almost all participants said they had changed their behavior, although some indicated that they needed to continue working at their complaints to control them. Since the perceived effects of the intervention generally met the factors of the I-Change model (2.0), it appears that the factors considered important for behavioral change were at least partly addressed by the intervention. However, on most of the outcome measures of the RCT (including self-efficacy), no significant difference was found between the intervention group and the usual care group at follow-up. Self-efficacy was already high, indicating a possible ceiling effect. Also, the discrepancy between the high satisfaction level of the participants in this study and the limited results of the RCT might also be caused (in part) by the extra attention received during the intervention leading to a high level of satisfaction. Moreover, during the interviews, participants who were positive about the intervention might have been giving ‘desired’ answers. However, since criticism was also given, and because the positive results of the interviews were confirmed by the questionnaire, this does not seem to be the case.

This study has several strengths. The use of an interview guide, respondent validation, and consensus coding of two authors ensured the validity of the results. Moreover, the results of the semi-structured interviews were accompanied and supported by a quantitative evaluation at three 12-months follow-up. Combining qualitative and quantitative research methods is common in social science [43] and qualitative evaluation of intervention programs is often performed [43, 45–50]. The use of qualitative methods can make an important contribution to the results of RCTs evaluating complex health service interventions [51, 52].

Most participants stated that the sessions and the eHealth module complemented each other, as was intended; however, some participants clearly preferred the sessions while others preferred the eHealth module. Given the variation in participant preferences, it seems that the combination of sessions *and* the eHealth module is a strength of the program. With regard to the implementation: future sessions might be more tailored to the needs of the group (e.g., more physical activity in the sessions); this was not possible in this intervention, because the moderators had to strictly follow the training protocol. In the future, a computer-tailored online program might better address these different needs of participants, and an online community might be used for the social interaction of participants. Eventually, the program might also be developed for a broader group of participants, e.g. for employees with work-related musculoskeletal disorders. A recent review showed that eHealth in somatic diseases is effective/cost-effective and that the evidence is at least promising [53].

This study also has imitations. Participants were only interviewed about (the expectations of) the program after the sessions and not before the program. Therefore, the participants’ expectations of the program could be influenced by their experiences during their participation. Moreover, the themes that emerged during the thematic analysis were influenced by the question guide of the semi-structured interviews.

Most participants worked in a hospital or educational setting and had a relatively high level of education. Although we found no major differences between the experiences of participants working in different environments, the experiences of employees with CANS might vary between different work environments. Also, because the interviews were held shortly after the last session and, although participants mentioned changes in their awareness, attitude and behavior, it remains unclear whether behavioral change was achieved on the long term. In addition, this study only included the participants’ perspective, whereas Including the perspectives of other stakeholders (e.g. colleagues, supervisors, and moderators) could have been valuable.

There was some criticism regarding aspects of the intervention itself. Some participants had expected a more physical approach during the intake and sessions, even though the information leaflet described the content of the self-management program; however, most of them still acknowledged the value of the psycho-social orientated approach. The need of such an approach is endorsed by earlier interviews with experts on self-management for employees with CANS [24] and by other research indicating that CANS interventions should not be restricted to ergonomic improvements, but should be accompanied by improvement of the job design from a psychological and social perspective [54]. The lack of a follow-up session is also a limitation, as many participants indicated that some kind of follow-up would be valuable for them. Therefore, a follow-up session should be included in future programs.

Although the eHealth module (especially the exercises) was generally experienced as positive, it was revealed that the eHealth module was not used extensively by all participants. This could be a major limitation to the implementation of this part of the intervention. Also, some parts of the eHealth module were used more than others (e.g. the physical exercises). In the quantitative evaluation 32.1 % of the participants indicated that they had not used the eHealth module in the first three months of the intervention [34]. Given that 76.9 % did use the eHealth module [34], it can be concluded that the eHealth was not valuable or usable for all participants. On the other hand, because our participants had CANS, they may not have intensively used the eHealth module out of fear that this might aggravate their complaints. Also, eHealth may not be the best way to provide information for all people, and personal preferences may well play a role. In our study, not all participants seemed aware of all the topics on the eHealth module, because the physical aspects of the complaints were sometimes mentioned as ‘missing topics’ but were in fact addressed in the eHealth module. Some people might need additional facilitation/support to use all the course materials to achieve the desired behavior. Therefore, it seems advisable to make a clearer referral to the more physically-orientated modules of the eHealth in the sessions or, for example, to include some physical aspects in each session, as was done in a self-management program for people with chronic pain [55]. Moreover, a computer-tailored eHealth module with content based on the participants’ characteristics, which leads participants through all the important topics, might be valuable.

In conclusion, participants of a self-management program, consisting of self-management sessions and an eHealth module, were satisfied with the program and most experienced benefit from the program. The results of the semi-structured interviews were supported by the quantitative evaluation which also showed a high level of satisfaction. These experiences and recommendations of the participants can be used to adapt and further implement the self-management program for employees with CANS and to develop other interventions for patients with CANS, or self-management programs for other musculoskeletal disorders.

## Electronic supplementary material

Below is the link to the electronic supplementary material.

Supplementary material 1 (DOCX 12 kb)

Supplementary material 2 (DOCX 16 kb)
