# Supplementary material for: Experiences of Participants in a Self-Management Program for Employees with Complaints of the Arm, Neck or Shoulder (CANS): A Mixed Methods Study
Source: J Occup Rehabil. 2016 Feb 13;27(1):35–48. doi: 10.1007/s10926-016-9630-9 (PMC5306216; doi:10.1007/s10926-016-9630-9)
Supplement: Supplementary file 1 — Supplementary material 1 (DOCX 12 kb) [file 10926_2016_9630_MOESM1_ESM.docx]

Appendix 1: Interview guide

1. Can you tell your personal reason to participate in the self-management program?
2. What were your expectations of the self-management program?
3. Fulfilled the self-management program your expectations? Why (not)?
4. Can you tell how you have benefitted from the self-management program?
5. Has the way you are looking at your complaints changed during the self-management program?
6. Do you have the confidence that you can deal with your complaints in the future? Why (not)?
7. Have you experienced any difficulties with performing the weekly action plans?
8. Do you think that the self-management sessions and the eHealth module were complementary to each other? Why (not)?
9. Did you miss any topics in the self-management program, or were there any superfluous topics?
10. Dou you have any recommendations for us to improve the self-management sessions?
11. Do you have any recommendations for us to improve the eHealth module?
