# Supplementary material for: Experiences of Participants in a Self-Management Program for Employees with Complaints of the Arm, Neck or Shoulder (CANS): A Mixed Methods Study
Source: J Occup Rehabil. 2016 Feb 13;27(1):35–48. doi: 10.1007/s10926-016-9630-9 (PMC5306216; doi:10.1007/s10926-016-9630-9)
Supplement: Supplementary file 2 — Supplementary material 2 (DOCX 16 kb) [file 10926_2016_9630_MOESM2_ESM.docx]

| Appendix 2: Demographic profile of the study population of the semi-structured interviews | | | | | | | | | | | | | | | |
| --- | --- | --- | --- | --- | --- | --- | --- | --- | --- | --- | --- | --- | --- | --- | --- |
| **ParticipantID number** | **Gender** | **Age (years)** | **Education level** | **Profession** | **Hours of work per week** | **Hours on PC**  **per day** | **Region of complaints** | | | | | | | **Duration of complaints (weeks)** | **Disability score on work (0-10)*** |
|  |  |  |  |  |  |  | **N** | **S** | **U1** | **E** | **U2** | **W** | **H** |  |  |
| 1 | female | 48 | HPE | Information specialist | 33-40 | 8 | + | + | + |  | + |  | + | 24 | 7 |
| 2 | female | 50 | HPE | Administrative assistant | 20-32 | 3 |  | + |  | + |  | + | + | 36 | 3 |
| 3 | female | 56 | SSVE | Administrative assistant | 20-32 | 6 | + | + |  |  |  |  |  | 60 | 0 |
| 4 | female | 41 | HPE | Counsellor education | 20-32 | 7 | + | + | + | + |  | + | + | 12 | 5 |
| 5 | female | 58 | PSE/UPE | Administrative assistant | 20-32 | 8 | + | + | + | + | + | + | + | 13 | 2 |
| 6 | female | 52 | HPE | Senior analyst in vitro fertilization | 20-32 | 1 | + | + | + |  | + | + | + | 52 | 1 |
| 7 | female | 48 | SSVE | Employee processing blood | 20-32 | 4 | + | + |  |  |  |  |  | 200 | 9 |
| 8 | female | 35 | SSVE | Staff planner | 20-32 | 8 | + | + | + |  |  |  |  | 234 | 4 |
| 9 | male | 54 | PSE/UPE | Application management ICT | 33-40 | 8 |  |  |  | + | + |  |  | 650 | 0 |
| 10 | female | 50 | SSVE | Neonatal intensive care nurse | 20-32 | 2 |  |  |  |  | + | + | + | 24 | 5 |
| 11 | female | 40 | SSVE | Receptionist, service desk employee | 20-32 | 8 | + | + | + |  |  | + | + | 100 | 5 |
| 12 | female | 54 | SSVE | Senior secretary complaints mediation | 20-32 | 8 |  | + | + |  | + | + |  | 16 | 5 |
| 13 | female | 56 | PSCE | Nursing- and nutrition assistant | 20-32 | 1 | + | + | + |  | + |  | + | 30 | 4 |
| 14 | female | 61 | PSE/UPE | Administrative assistant | 33-40 | ? | + | + |  |  |  | + |  | 20 | 4 |
| 15 | male | 29 | HPE | Dental hygienist | 20-32 | 12 | + | + |  |  |  | + | + | 26 | 4 |
| 16 | female | 47 | AHE | Research grant advisor | 33-40 | 8 | + | + |  |  | + |  | + | 69 | 3 |
| 17 | female | 30 | AHE | PhD student | >40 | 8 | + | + |  |  |  |  |  | 150 | 5 |
| 18 | female | 53 | PSE/UPE | Secretary | 20-32 | 8 | + | + | + |  | + |  | + | 60 | 3 |
| 19 | female | 60 | HPE | Administrative assistant | 20-32 | ? | + | + |  |  |  | + |  | 12 | 0 |
| 20 | female | 39 | AHE | Paediatrician | >40 | 0 | + | + | + | + | + | + | + | 20 | 8 |
| 21 | male | 43 | PSCE | Controller supervision gambling | 33-40 | 2 | + | + | + |  | + |  |  | 30 | 0 |
| 22 | female | 56 | HPE | Painter (artist) | 33-40 | ? | + |  | + | + | + |  |  | 12 | 3 |
| 23 | female | 52 | SSVE | Analyst biochemical laboratory | 33-40 | 7 | + | + | + |  | + |  |  | 16 | 2 |
| 24 | female | 28 | AHE | Desk editor at local broadcasting | 12-19 | 9 | + | + | + | + |  |  |  | 208 | 4 |
| 25 | male | 50 | HPE | Warranty analyst | 33-40 | 8 |  | + | + | + | + | + |  | 20 | 1 |
| 26 | female | 56 | HPE | Photography, journalist, writing | 20-32 | 5 |  | + | + | + | + |  |  | 520 | 3 |
| 27 | female | 29 | AHE | PhD student | 33-40 | 8 | + | + |  |  |  |  |  | 32 | 4 |
| 28 | female | 27 | AHE | Text writing, journalist | 20-32 | ? | + | + | + | + | + | + | + | 168 | 4 |
| 29 | male | 28 | AHE | PhD student | >40 | 9 | + | + | + | + | + |  |  | 208 | 7 |
| 30 | female | 56 | PSE/UPE | Secretary | 33-40 | 6 |  | + | + |  | + |  | + | 624 | 7 |
| 31 | female | 44 | HPE | Assistant general practitioner | 12-19 | 2 | + | + |  |  | + |  | + | 72 | 5 |
| PSCE = Preparatory secondary vocational education, SSVE = Senior secondary vocational education, PSE/UPE = Prevocational secondary education / University preparatory education, HPE = Higher professional education, AHE = Academic higher education, N = Neck, S = Shoulder, U1 = upper arm, E = Elbow, U2 = Under arm, W = Wrist, H = Hand,  *A score of 10 means completely disabled | | | | | | | | | | | | | | | |
